# Disclosing the Overall and Sex‐Related Disease Burden and Treatment Needs of Patients With Anogenital Psoriasis: Results From the Case–Control PsoGen Study

**DOI:** 10.1155/drp/7159192

**Published:** 2026-08-13

**Authors:** Neuza da Silva Burger, Matthias Augustin, Dagmar Wilsmann-Theis, Athanasios Tsianakas, Petra Staubach-Renz, Rachel Sommer

**Affiliations:** ^1^ German Center for Health Services Research in Dermatology (CVderm), Institute for Health Services Research in Dermatology and Nursing (IVDP), University Medical Center Hamburg-Eppendorf, Hamburg, Germany, uke.de; ^2^ Department of Dermatology and Allergology, University Medical Center Bonn, Bonn, Germany; ^3^ Department of Dermatology, Specialist Clinic Bad Bentheim, Bad Bentheim, Germany; ^4^ Department of Dermatology, University Medical Center Mainz, Mainz, Germany, unimedizin-mainz.de

**Keywords:** anogenital psoriasis, patient-defined treatment needs and benefits, quality of life, sexual impairments, social stigma

## Abstract

**Purpose:**

Psoriasis lesions in the anogenital area affect about 33%–63% of the patients at some point in the disease course. Diminished quality of life (QoL), high depression rates, and sexual impairments have been described among patients with anogenital psoriasis, but specific treatment needs of these patients are often neglected in routine healthcare. This study aimed to compare patients with vs. without current anogenital lesions in terms of their sociodemographic and clinical characteristics, patient‐reported outcomes (PROs) of disease burden, and patient‐defined treatment needs/benefits and to identify clinical variables associated with PROs of disease burden.

**Patients and Methods:**

The study had a cross‐sectional case–control design. Patients aged ≥ 18 years with psoriasis were consecutively recruited in four German dermatology centers and were assigned to case or control group based on both patients’ and physicians’ reports of current anogenital involvement. PROs included the assessment of QoL impairments, treatment needs/benefits, depression/anxiety, perceived stigmatization, sexual frequency, and relationship/sexual impairments. Physicians assessed the severity of psoriasis and anogenital lesions (PASI and sPGA‐G).

**Results:**

Participants were 294 patients with psoriasis, of which 159 (54.1%) had current anogenital lesions. Patients with anogenital lesions had higher disease severity, more comorbidities, but were less often treated with biologics. They also reported more QoL impairments, less treatment benefits, poorer mental health, and more sexual impairments. Specific treatment needs of patients with anogenital psoriasis were related to reducing physical and relationship/sexual impairments. Poorer PROs were explained at 25%–68% by higher disease severity, anogenital lesions, no biologic treatment, higher intensity of itching/burning, and lower frequency and more limitations in sexual activity.

**Conclusion:**

Within a patient‐centered approach to dermatology care for psoriasis, clinical decisions, particularly patients’ qualification for treatment with biologic drugs, should consider not only the location, extent, and severity of psoriasis lesions but also patient‐reported symptoms, overall disease burden, and sexual limitations.

## 1. Introduction

Psoriasis is a chronic inflammatory dermatological condition that affects about 2%–4% of the population in Western countries and 2.5% in Germany [1, 2]. Genital psoriasis is a well‐known though less common form, which, in its isolated form, has an estimated prevalence of 2%–5% of all patients with psoriasis [3]. However, 33%–63% of the patients with different psoriasis phenotypes experience psoriatic lesions in the anal and/or genital areas at some point during the course of the disease [4].

The disease burden of anogenital psoriasis is high, with patients presenting poor health, mental health, and psychosocial outcomes [5]. A systematic review of 14 studies revealed significantly diminished quality of life (QoL), higher levels of stigmatization, and more depressive symptoms among patients with genital psoriasis, compared with patients with psoriasis without genital involvement [6]. In another study on the topology of psoriasis, the genital involvement was identified as a significant predictor of decreased QoL [7]. In addition, both the physical symptoms of anogenital psoriasis (e.g., cracked skin and pain or discomfort) and the feelings of embarrassment in exposing the affected areas often result in avoidance or severe impairments in intimate relationships and sexual activity [8, 9].

The significant QoL impairments of patients with anogenital psoriasis qualify them for systemic therapy, independently of the overall extent of affected body surface area (BSA) [10, 11]. However, anogenital lesions often remain undisclosed because of embarrassment and discomfort about discussing sex‐related issues during healthcare visits [5, 12]. In addition, clinical decisions in routine care are often based on the Psoriasis Area and Severity Index (PASI) [13] and Dermatology Life Quality Index (DLQI) [14] scores. However, the PASI is unable to capture the severity of anogenital lesions [15]. In the DLQI, more than 10% of the patients tend to rate the sex‐related item as 0 = “not relevant.” These “not relevant” responses are coded as zero, meaning “no impairments in sex life.” However, these “not relevant” responses can also indicate a complete absence of sex life because of the illness (i.e., maximal impairment in sex life); in these cases, the DLQI score underestimates the patients’ disease burden [16, 17]. Therefore, anogenital psoriasis remains underdiagnosed in a significant percentage of patients and their specific treatment needs and goals are often neglected in the clinical decision.

In this context, the present study assessed a broad range of patient‐reported outcomes (PROs) of overall and sex‐related disease burden in patients with psoriasis, with and without lesions in the anogenital areas. Specific aims were to (1) compare patients with and without current anogenital lesions in terms of their sociodemographic and clinical characteristics, PROs of disease burden, and treatment benefits; (2) identify specific, and often hidden, needs regarding psoriasis management of patients with anogenital psoriasis, in comparison to those without anogenital lesions; and (3) test the associations between sociodemographic and clinical characteristics and PROs of disease burden and treatment benefits.

## 2. Material and Methods

### 2.1. Study Design and Participants

This cross‐sectional observational case–control study was approved by the Ethics Committee of the Hamburg Chamber of Physicians (PV7160, December 10, 2019) and was conducted following the principles of the World Medical Association Declaration of Helsinki (1964, and its later amendments). Using the nonprobabilistic convenience sampling method, participants were recruited between April 2020 and October 2022 in four German centers in Hamburg, Bonn, Bad Bentheim, and Mainz. Patients were initially screened by their physician and those who (1) were aged ≥ 18 years, (2) had a clinical diagnosis of moderate‐to‐severe psoriasis, based on PASI scores ≥ 10 and BSA ≥ 3%, (3) were able to follow the instructions of the study and to complete the questionnaires in German language, and (4) signed the Informed Consent Form (ICF) were included in the study.

For comparative analyses, patients were assigned to case or control group based on a combination of two clinician‐reported and two patient‐reported indicators of current anogenital involvement, according to the following definitions:

• Patients with current anogenital psoriasis (cases): the physician reported current anogenital involvement in a yes/no question and assessed the severity of anogenital lesions with Static Physician’s Global Assessment of Genitalia (sPGA‐G) ≥ 1; and the patient reported current anogenital involvement in a yes/no question or marked at least one square in the genital area (front view) or anal area (back view) in the topology grid.
• Patients with psoriasis without anogenital involvement (controls): the physician reported no current anogenital involvement in a yes/no question and assessed the severity of anogenital lesions with sPGA‐G  ≤ 1; and the patient reported no current anogenital involvement in a yes/no question or did not mark any squares in the anogenital area in the topology grid.

The participants for whom the patient and physician reports were completely discrepant and could not be clearly assigned to the case or control group were excluded.

### 2.2. Outcomes Measures

For each participant, two sets of questionnaires were completed, one by the physician and one by the patient. The physicians provided information about disease characteristics, including current involvement of genitalia, treatments, and comorbidities; the percentage of BSA affected by redness, thickness, and scaling (score range from 0% to 100%, with BSA between 3% and 10% considered as moderate and BSA > 10% considered as severe psoriasis) [18]; the overall disease severity (PASI; score range from 0 to 72 with scores ≥ 10 considered as moderate‐to‐severe psoriasis) [13]; and the severity of genital psoriasis in terms of three plaque characteristics of erythema, elevation, and scale (sPGA‐G; single score from 0 = *clear* to 5 = *very severe*) [19].

The patients provided sociodemographic information, reported on current involvement of genital and/or anal body areas by answering a yes/no question and by reporting on the topical distribution of psoriasis using a high‐resolution grid scheme with 1424 small squares [7], and assessed the intensity of psoriasis symptoms of pruritus, pain, and burning sensations using a 0–10 numeric rating scale (NRS). In addition, the patients completed the German versions of the following standardized PROs: DLQI [14], Patient Benefit Index (PBI) [20], Patient Health Questionnaire (PHQ‐4) [21], Perceived Stigmatization Questionnaire (PSQ) [22, 23], Relationship and Sexuality Scale (RSS) [24, 25], and Genital Psoriasis Sexual Frequency Questionnaire (GenPs‐SFQ) [8]. A summarized description of these questionnaires, as well as their internal consistency (Cronbach’s alpha) in the current sample, is provided in Table 1.

**TABLE 1:** Summary of patient‐reported outcomes (PROs) and reliability (Cronbach’s alpha).

PROs		Construct assessed	*N*. of items	Response scale	Scoring and interpretation	Reliability
Dermatology Life Quality Index (DLQI)		Skin‐generic quality of life	10	0 = “not at all”/“not relevant” to 3 = “very much”	Sum score ranging from 0 to 30; higher scores indicate larger quality of life impairments	0.93

Patient Benefit Index (PBI)	Patient Needs Questionnaire (PNQ)	Importance of individual needs	25	0 = “not at all”/“does not apply to me” to 4 = “very”	PBI computed from the arithmetic mean of all rated benefits (PBQ items) weighted by the relative importance of each corresponding PNQ item; range from 0 (no benefit) to 4 (maximal benefit)	0.93
	Patient Benefits Questionnaire (PBQ)	Perceived benefits from treatments	25	0 = “not at all”/“did not apply to me” to 4 = “very”		0.97

Patient Health Questionnaire (PHQ‐4)	Patient Health Questionnaire (PHQ‐2)	Screening for depression disorders	2	0 = “not at all” to 3 = “nearly every day”	Composite sum score ranging from 0 to 12; higher scores indicate worse mental health	0.88
	Generalized anxiety disorder screener (GAD‐2)	Screening for anxiety disorders	2			

Perceived Stigmatization Questionnaire (PSQ)		Social experiences of people with appearance distinctions, in terms of their perceptions of others’ confused/staring behaviors, absence of friendly behaviors, and hostile behaviors	21	0 = “never” to 4 = “always”	Sum score ranging from 0 to 84; higher scores indicate higher levels of perceived stigmatization	0.90

Relationship and Sexuality Scale (RSS)		Impact of a disease and its treatments on sexual function, sexual frequency, and sexual fear	10	0–4 (items 1, 4, 5, 6, 9, 10) or 0–3 (items 2, 3, 7, 8)	Sum score ranging from 0 to 36; higher scores indicate more sexual impairments	0.79

Genital Psoriasis Sexual Frequency Questionnaire (GenPs‐SFQ)		Frequency of sexual activity	1	0 = “none”; 1 = “once”; 2 = “twice or more”	Items are analyzed individually	—
		Limitation in the frequency of sexual activity because of genital psoriasis	1	0 = “never” to 4 = “always”		

### 2.3. Statistical Analyses

The statistical analyses were performed with the IBM SPSS Statistics for Windows (SPSS, V.29.0.1.0, IBM Corp., Armonk, NY). Statistical significance was set at *p* value < 0.05. Missing data on PROs that were random and < 15% of the values were replaced with the individual mean score for each variable. Descriptive statistics were obtained for sociodemographic and clinical variables, as well as for PROs of disease burden. The homogeneity of sample characteristics between the groups of patients with and without anogenital lesions was examined by independent samples *t*‐tests (continuous variables) or chi‐squared tests (categorical variables).

PROs of disease burden were compared between the groups of patients with and without anogenital involvement using univariate analysis of covariance (ANCOVA). Covariates included were sociodemographic and disease characteristics that significantly differed between the groups and were at least weakly correlated (Pearson *r* ≥ 0.20) with the outcome variable [26]. Effect size were presented for the comparison analyses, considering ${ŋ}_{p}^{2}$ ≥ 0.01 as small effect, ${ŋ}_{p}^{2}$ ≥ 0.06 as medium effect, and ${ŋ}_{p}^{2}$ ≥ 0.14 as large effect [27]. Additionally, differences in specific patient needs (PNQ items) were tested with nonparametric Mann–Whitney *U* tests.

Hierarchical linear regression analyses were performed to identify (1) sociodemographic variables, (2) disease characteristics, and (3) patient‐reported symptoms and limitations associated with PROs of disease burden and treatment benefits. In the multivariable models, variance inflation factors (VIFs) > 5 were considered as indicators of multicollinearity problems [28].

## 3. Results

### 3.1. Patient’s Sociodemographic and Clinical Characteristics

A total of 343 patients were recruited. Of these, 10 questionnaires were not returned to the coordinating center and 13 questionnaires had missing values in a percentage higher than 15% and were excluded. In addition, 26 patients were excluded because their anogenital involvement could not be clearly determined. The final sample was, therefore, composed by 294 patients, with 159 patients (54.1%) assigned to the group of cases with psoriasis involving the anogenital area and 135 patients (45.9%) defined as controls. Table 2 displays the descriptive statistics for sociodemographic and clinical characteristics of patients with and without anogenital psoriasis and the comparative analyses between the groups.

**TABLE 2:** Sociodemographic and clinical characterization of patients with psoriasis with and without anogenital involvement.

		Anogenital psoriasis	No anogenital involvement	*X* ^2^/*t*	*p*
*Sociodemographic characteristics*					
Sex, *n* (%)	Male	102 (64.2)	94 (69.6)	0.99	0.321
	Female	57 (35.8)	41 (30.4)		

Age, *M* ± SD		42.80 ± 13.45	43.17 ± 14.79	0.23	0.822

*Disease characteristics*					
Type of psoriasis, *n* (%)^a^	Plaque‐type psoriasis	147 (92.5)	131 (97.0)	2.40	0.122
	Intertriginous psoriasis	103 (64.8)	18 (13.3)	80.75	< 0.001
	Guttate psoriasis	22 (13.8)	15 (11.1)	0.52	0.470
	Pustular psoriasis	6 (3.8)	4 (3.0)	0.15	0.695
	Psoriatic arthritis	50 (32.1)	29 (21.5)	4.28	0.039

%BSA, *M* ± SD		9.36 ± 11.16	3.44 ± 8.08	−5.04	< 0.001
	Missing, *n* (%)	12 (7.5)	2 (1.5)		

PASI, *M* ± SD		7.60 ± 6.92	2.39 ± 4.21	−7.63	< 0.001

Disease duration (years), *M* ± SD		18.89 ± 13.80	20.44 ± 14.20	0.94	0.349
	Missing, *n* (%)	2 (1.3)	4 (3.0)		

Systemic treatment, *n* (%)^a^	Biologic	71 (44.7)	100 (74.1)	25.97	< 0.001
	Conventional	41 (25.8)	17 (12.6)	8.03	0.005

Comorbidities (yes), *n* (%)		100 (62.9)	63 (46.7)	7.78	0.005

*Patient-reported symptoms and limitations*					
Intensity of pain^b^, *M* ± SD		3.53 ± 3.01	1.02 ± 1.97	−8.24	< 0.001
	Missing, *n* (%)	0 (0.0)	2 (1.5)		

Intensity of itching^b^, *M* ± SD		5.57 ± 2.85	2.26 ± 2.84	−9.89	< 0.001
	Missing, *n* (%)	0 (0.0)	2 (1.5)		

Intensity of burning^b^, *M* ± SD		4.17 ± 3.13	1.47 ± 2.48	−8.04	< 0.001
	Missing, *n* (%)	0 (0.0)	3 (2.2)		

Sexual frequency, *n* (%)^c^	None	69 (43.4)	42 (31.1)	2.84	0.092
	Once/Twice or more	84 (52.8)	78 (57.8)		
	Missing	6 (3.8)	15 (11.1)		

Limitation in sexual activity, *n* (%)^d^	Never/Rarely	80 (50.3)	112 (83.0)	57.82	< 0.001
	Sometimes/Often/Always	72 (45.3)	6 (4.4)		
	Missing	7 (4.4)	17 (12.6)		

Abbreviations: BSA, body surface area; PASI, Psoriasis Area and Severity Index.

^a^Multiple answers were possible and, thus, no cumulative % can be calculated.

^b^Patient reported by using a 0–10 numeric rating scale (NRS).

^c^GenPS‐SFQ Item 1: In the past week, how many times did you engage in sexual activity?

^d^GenPS‐SFQ Item 2: In the past week, how often did your genital psoriasis limit the frequency of your sexual activity?

The groups of patients with and without anogenital involvement were homogeneous in terms of sociodemographic characteristics. However, patients with anogenital psoriasis were more often diagnosed with intertriginous psoriasis and psoriatic arthritis, presented higher disease severity but were less often treated with biologic drugs, and had more often comorbid conditions. In addition, patients with anogenital psoriasis reported higher intensity of symptoms of pain, pruritus, and burning sensation, and, although there were no significant differences in the frequency of sexual activity, they reported more limitations in sexual activity because of psoriasis.

Regarding specific clinical characterization of patients with anogenital psoriasis, 72 patients (45.3%) were affected in the genital area alone, 16 (10.1%) in the anal area alone, and 70 (44.0%) were affected in both the genital and anal areas. These patients had anogenital psoriasis, in average, for 7.67 ± 10.88 years, and the severity of anogenital lesions, as assessed by the sPGA‐G, was 2.32 ± 0.95.

### 3.2. PROs of Disease Burden and Patient‐Defined Treatment Needs

Preliminary correlation analyses showed that DLQI impairments were moderately correlated with higher PASI (*r* = 0.53) and weakly correlated with no biologic therapy (*r* = −0.33); patient benefits (PBI) were moderately correlated with biologic treatment (*r* = 0.48) and weakly correlated with lower PASI (*r* = −0.32) and no conventional systemic treatment (*r* = −0.25); worse mental health (PHQ‐4) was weakly correlated with the diagnosis of intertriginous psoriasis (*r* = 0.22), higher PASI (*r* = 0.34), and no biologic therapy (*r* = −0.29); perceived stigmatization (PSQ) was weakly correlated with higher PASI (*r* = 0.32); and sexual impairments were weakly correlated with the diagnosis of intertriginous psoriasis (*r* = 0.29) and higher PASI (*r* = 0.35). These clinical variables were included as covariates in the comparison analyses for the respective PROs of disease burden, while acknowledging that statistical adjustment may not fully account for overlap between overall disease severity and anogenital involvement.

The analyses of covariance (Table 3) showed that patients with anogenital psoriasis reported more QoL impairments, poorer mental health, less patient‐defined treatment benefits, and more sexual impairments. No significant differences were found for the levels of perceived stigmatization.

**TABLE 3:** Comparative analyses of PROs of disease burden between patients with and without anogenital involvement.

	Anogenital psoriasis		No anogenital involvement		*F*	*p*	${ŋ}_{p}^{2}$
	*n*	*M* ± SD	*N*	*M* ± SD			
QoL impairments (DLQI)	156	10.87 ± 7.29	134	3.64 ± 5.38	32.69	< 0.001	0.103
Patient benefits (PBI)	116	2.02 ± 1.24	113	3.14 ± 1.06	24.95	< 0.001	0.100
Mental health (PHQ‐4)	156	3.86 ± 3.00	133	1.74 ± 2.67	7.86	0.005	0.027
Perceived stigmatization (PSQ)	153	24.05 ± 12.97	133	18.65 ± 12.66	2.19	0.140	0.008
Sexual impairments (RSS)	146	18.96 ± 6.50	126	13.88 ± 4.91	13.21	< 0.001	0.047

Abbreviations: DLQI, Dermatology Life Quality Index (range: 0–30); PBI, Patient Benefit Questionnaire (range: 0–4); PHQ, Patient Health Questionnaire (range: 0–12); PSQ, Perceived Stigmatization Questionnaire (range: 0–84); QoL, quality of life; RSS, Relationship and Sexuality Scale (range: 0–36).

**FIGURE 1:**
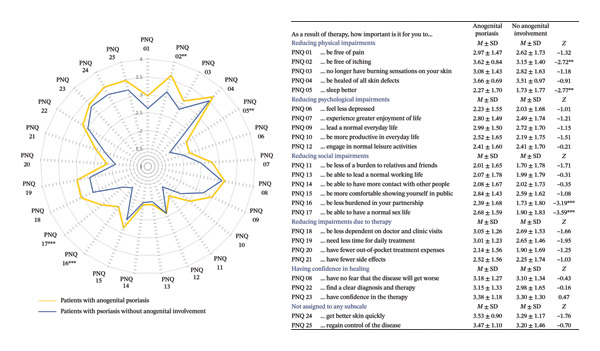
Specific patient needs of patients with anogenital psoriasis and patients with psoriasis not involving the anogenital body areas. *Note: Z*, Wilcoxon signed rank test; ^∗^ *p* ≤ 0.050, ^∗∗^ *p* ≤ 0.010, ^∗∗∗^ *p* ≤ 0.001, two‐tailed. Abbreviation: PNQ, Patient Needs Questionnaire (range: 0–4).

Regarding specific patient needs, similar patterns were observed for patients with and without anogenital involvement (Figure 1). For all patients, the most relevant patient needs were “to be healed of all skin defects” (92.4% of patients rated as very important or quite important), “to get better skin quickly” (86.2%), “to be free of itching” (85.9%), “to regain control of the disease” (85.2%), and “to have confidence in the therapy” (84.4%). Nevertheless, patients with anogenital psoriasis reported significantly higher needs related to “be free of itching,” “sleep better,” “be less burdened in your partnership,” and “be able to have a normal sex life,” when compared to patients with psoriasis without involving the anogenital area (Figure 1).

### 3.3. Associations Between Sociodemographic Variables, Disease Characteristics, Patient‐Reported Symptoms and Limitations, and PROs of Disease Burden and Treatment Benefits

The hierarchical linear regression models testing the effects of sociodemographic variables, disease characteristics, and patient‐reported symptoms on PROs of disease burden are summarized in Table 4. The regression model for DLQI explained 68% of its variance, with the presence of anogenital lesions, higher disease severity, presence of comorbidities, higher intensity of itching and burning sensations, and lower frequency and greater limitations in sexual activity correlating significantly with more QoL impairments. Greater patient‐defined treatment benefits were explained at 46% by no involvement of anogenital body areas, prescription of biologic and no conventional systemic treatment, and lower intensity of itching. The variance in patients’ mental health was explained at 38%, with more symptoms of depression and anxiety associated with female sex, anogenital involvement, higher PASI, no biologic treatment, higher intensity of burning sensations, and more limitations in sexual activity because of psoriasis. Higher disease severity and more limitations in sexual activity because of psoriasis were also associated with higher levels of perceived stigmatization, explaining 25% of its variance. The final model explained 54% of the variance in sexual and relationship impairments; specifically, more impairments were associated with anogenital involvement, higher PASI, and lower frequency and more limitations in sexual activity because of psoriasis. These models were not limited by multicollinearity problems since all VIF were < 5 (across all models, max. VIF was 4.11 for “intensity of itching”).

**TABLE 4:** Regression analyses explaining the variance in PROs of disease burden and treatment benefits.

	QoL impairments (DLQI)		Patient benefits (PBI)		Mental health (PHQ‐4)		Perceived stigmatization (PSQ)		Sexual impairments (RSS)	
	*ß*	*t*	*ß*	*t*	*ß*	*t*	*ß*	*t*	*ß*	*t*
Sociodemographic characteristics	Δ*R* ^2^ = 0.01; Δ*F*_(2, 254)_ = 0.87		Δ*R* ^2^ = 0.01; Δ*F*_(2, 200)_ = 1.27		Δ*R* ^2^ = 0.03; Δ*F*_(2, 254)_ = 3.85^∗^		Δ*R* ^2^ = 0.01; Δ*F*_(2, 251)_ = 1.36		Δ*R* ^2^ = 0.02; Δ*F*_(2, 248)_ = 2.38	

Sex^a^	0.08	1.23	−0.04	−0.51	0.16	2.63^∗∗^	0.02	0.32	0.07	1.18

Age	−0.02	−0.35	0.10	1.48	−0.04	−0.65	−0.10	−1.58	0.12	1.92

Disease characteristics	Δ*R* ^2^ = 0.42; Δ*F*_(6, 248)_ = 30.25^∗∗∗^		Δ*R* ^2^ = 0.38; Δ*F*_(6, 194)_ = 19.92^∗∗∗^		Δ*R* ^2^ = 0.20; Δ*F*_(6, 248)_ = 10.68^∗∗∗^		Δ*R* ^2^ = 0.15; Δ*F*_(6, 245)_ = 7.38^∗∗∗^		Δ*R* ^2^ = 0.23; Δ*F*_(6, 242)_ = 12.05^∗∗∗^	

Anogenital involvement	0.24	4.28^∗∗∗^	−0.30	−4.51^∗∗∗^	0.19	2.95^∗∗^	0.09	1.38	0.30	4.62^∗∗∗^

PASI	0.39	7.21^∗∗∗^	−0.07	−1.11	0.18	2.79^∗∗^	0.34	5.06^∗∗∗^	0.26	4.08^∗∗∗^

Disease duration	−0.06	−1.00	0.08	1.15	−0.06	−0.94	0.09	1.30	−0.11	−1.71

Biologic treatment^b^	−0.11	−1.96	0.29	4.52^∗∗∗^	−0.16	−2.45^∗^	0.01	0.17	0.03	0.52

Conventional systemic treatment^b^	0.08	1.63	−0.17	−2.81^∗∗^	0.06	0.93	−0.06	−0.89	−0.04	−0.72

Comorbidities^b^	0.14	2.65^∗∗^	−0.10	−1.69	0.10	1.60	0.03	0.53	0.03	0.52

Patient‐reported symptoms and limitations	Δ*R* ^2^ = 0.26; Δ*F*_(5, 243)_ = 38.99^∗∗∗^		Δ*R* ^2^ = 0.08; Δ*F*_(5, 189)_ = 5.63^∗∗∗^		Δ*R* ^2^ = 0.15; Δ*F*_(5, 243)_ = 11.94^∗∗∗^		Δ*R* ^2^ = 0.08; Δ*F*_(5, 240)_ = 5.36^∗∗∗^		Δ*R* ^2^ = 0.30; Δ*F*_(5, 237)_ = 30.73^∗∗∗^	

Intensity of pain	0.04	0.58	−0.05	−0.58	0.09	1.10	0.01	0.15	−0.03	−0.43

Intensity of itching	0.20	2.78^∗∗^	−0.37	−3.49^∗∗∗^	0.12	1.16	0.05	0.44	0.15	1.65

Intensity of burning	0.32	4.53^∗∗∗^	0.06	0.63	0.21	2.17^∗^	0.15	1.40	−0.004	−0.05

Sexual frequency^c^	−0.08	−2.18^∗^	0.06	1.08	−0.01	−0.13	−0.03	−0.50	−0.44	−9.67^∗∗∗^

Limitation in sexual activity^d^	0.23	5.27^∗∗∗^	0.06	1.02	0.22	3.64^∗∗∗^	0.24	3.57^∗∗∗^	0.35	6.76^∗∗∗^

Model summary	*R* ^2^ = 0.68		*R* ^2^ = 0.46		*R* ^2^ = 0.38		*R* ^2^ = 0.25		*R* ^2^ = 0.54	
	*F*_(13, 243)_ = 40.05^∗∗∗^		*F*_(13, 189)_ = 12.80^∗∗∗^		*F*_(13, 243)_ = 11.50^∗∗∗^		*F*_(13, 240)_ = 6.03^∗∗∗^		*F*_(13, 237)_ = 21.54^∗∗∗^	

Abbreviations: DLQI, Dermatology Life Quality Index; PBI, Patient benefit questionnaire; PHQ, Patient health questionnaire; PSQ, Perceived Stigmatization Questionnaire; QoL, quality of life; RSS, Relationship and Sexuality Scale.

^a^0 = Male, 1 = Female.

^b^0 = No, 1 = Yes.

^c^0 = None, 1 = Once/Twice or more.

^d^0 = Never/Rarely, 1 = Sometimes/Often/Always.

^∗^ *p* ≤ 0.050.

^∗∗^ *p* ≤ 0.010.

^∗∗∗^ *p* ≤ 0.001, two‐tailed.

## 4. Discussion

This study contributed to better understanding the profiles of patients with anogenital psoriasis, in comparison to patients with psoriasis not involving the anogenital area, in terms of their sociodemographic and clinical characteristics, and also regarding a broad range of overall and sex‐related PROs of disease burden and treatment needs. Strengths of the study include the use of a combination of physician‐ and patient‐reported methods to determine the presence of anogenital lesions. This is especially important in anogenital psoriasis because many patients do not disclose their genital lesions to their physicians because of feelings of embarrassment or because they do not recognize the genital lesions part of the psoriasis clinical manifestations [12, 29]. Additionally, physicians do not always examine the genital area in routine appointments [30, 31], leading to a significant underestimation of the prevalence of anogenital psoriasis [5] and inaccuracies in results from case–control studies.

In terms of clinical parameters, there were significant differences between the two study groups, with patients with anogenital psoriasis presenting more often intertriginous psoriasis and psoriatic arthritis, larger %BSA affected by psoriasis, higher PASI, and more comorbidities, as well as higher intensity of patient‐reported symptoms of pain, itching, and burning sensations, and more limitations in sexual activity. This clinical characterization is consistent with previous studies identifying flexural involvement, concomitant involvement of other visible body areas, and higher disease severity as predisposing factors for the development of genital psoriasis [25, 32, 33]. Other studies have also described itching, pain, burning, and dyspareunia in a high portion of patients with anogenital lesions, particularly in female patients [25, 30, 33]. Additional commonly reported risk factors include male sex, early onset of psoriasis, and longer disease duration [25, 32], but these were not confirmed in our sample.

Comparative analyses of PROs of disease burden showed that patients with anogenital psoriasis reported more QoL impairments, poorer mental health, and more sexual and relationship impairments than patients with psoriasis not involving the anogenital areas. However, these results need to take into account that patients with anogenital psoriasis had higher overall disease severity in terms of PASI and BSA. Although PASI and other relevant covariates were included in adjusted analyses, residual confounding cannot be excluded. In particular, overall disease severity and anogenital involvement are likely to be correlated, making it difficult to fully disentangle the contribution of lesion location from that of more severe overall disease in a cross‐sectional study.

In addition to the location of psoriasis lesions in the anogenital areas, other clinical features that are more prevalent among these patients, such as higher PASI, presence of comorbidities, higher intensity of itching and burning sensations, and lower frequency and greater limitations in sexual activity because of psoriasis, were associated with higher disease burden. These results converge with the existing literature [6, 25, 29] and support the high burden associated with anogenital psoriasis, in general and specifically related to sexuality and intimate relationships. Regarding perceived stigmatization, no significant differences between the groups were found, suggesting that the social impairments caused by psoriasis might be more dependent on the visibility of the lesions than the involvement of sexually sensitive body areas [34–36]. This result may be due to the choice of measurement. The PSQ primarily assesses social stigmatization, while genital involvement is not visible to most people and, therefore, does not lead to increased social stigmatization. In the future, it would be important to use instruments that assess self‐stigmatization, as more pronounced differences between the two groups are likely to be found here, which could possibly also explain the differences in mental health.

Patients with anogenital psoriasis considered certain patient‐defined treatment needs to be particularly important. These included reducing itching and sleep problems, as well as improving impairments in sexual and relationship life. A wide range of treatment needs, and particularly related to reducing sexual impairment, were previously described for patients with anogenital psoriasis, in comparison with patients without anogenital lesions [29, 37]. Furthermore, patients with genital psoriasis reported less treatment benefits, which suggest that their high needs are not being fully addressed by current therapies. The gap between high needs and low benefits for this hard‐to‐treat area might be explained by the involvement of mechanical trigger factors, limited topical therapy choices, and the embarrassment of both patients and physicians in discussing sex‐related impairments and needs during routine appointments [30, 38–40].

Despite the higher disease severity and burden, patients with anogenital psoriasis were less often treated with biologic drugs. This could also be due to the abovementioned reason, of both patients and physicians feeling not comfortable examining sexually sensitive body areas and discussing sex‐related impairments, leading to undertreatment of this patient population. Besides, being treated with a biologic agent was associated with more patient‐defined treatment benefits and less mental health problems, while being treated with a conventional systemic drug was associated with less treatment benefits. A systematic review examining the efficacy, safety, and tolerability of topical and systemic treatments for genital psoriasis concluded that (low‐potency) corticosteroids are still recommended as first‐line treatment for genital psoriasis [41]. With the introduction of biologic drugs, several interleukin (IL)‐17 and IL‐23 inhibitors have demonstrated efficacy not only in achieving clearance of the genital lesions [42, 43] but also in reducing itching and limitations in sexual activity [44–46]. Therefore, clinical decisions, particularly the qualification of patients for therapy with biologics, should consider the location, extent, and severity of psoriasis lesions and the patient‐reported symptoms, overall burden, and sexual limitations.

Study limitations include the cross‐sectional design with simultaneous assessment of patient needs and benefits with regard to current treatment (or previous until date of assessment), thus excluding causal interpretations. Moreover, the nonprobabilistic convenience sampling method may have introduced selection bias, as patients presenting to specialized dermatology centers or willing to participate might differ systematically from the broader psoriasis population, thereby limiting the generalizability of the findings. As another study caveat, the number and duration of previous systemic treatment(s), and whether the anogenital lesions were discussed and taken into account in the clinical decision, were not documented. Prior or ongoing biologic treatment may have influenced baseline disease severity, potentially biasing comparisons between groups. For example, patients allocated to the nonanogenital group might have had anogenital psoriasis in the past. Although they do not have lesions anymore because of prior treatment, they still might suffer from persisting psychosocial burden. Another limitation was the modest sample size and the lower portion of female patients prevented further group splitting by patients’ sex. Finally, sexual activity and related impairments were assessed by self‐report. Because these are sensitive topics, some patients may have underreported difficulties due to embarrassment or social desirability, which could have resulted in conservative estimates of sex‐related disease burden. Further research is still necessary to identify additional psychosocial predictors and coping mechanism explaining the poor (mental) health outcomes that can be targeted in psychosocial interventions, in order to advocate for multidisciplinary healthcare of patients with anogenital psoriasis.

## 5. Conclusions

In summary, patients with anogenital psoriasis have higher disease burden related to both disease parameters (e.g., severity) and psychosocial outcomes (e.g., QoL, mental health, and sexual functioning) compared to patients with psoriasis without anogenital involvement. They also report specific treatment needs mostly related to reducing physical impairments (itching and sleep disturbances), as well as social impairments in their intimate and sexual relationships. By identifying overall and sex‐related impairments in PROs of disease burden, as well as specific needs of patients with anogenital lesions, this study provides new insights for optimized patient‐centered psoriasis management aiming both the achievement of skin clearance and maximized patient‐defined treatment benefits. Clinical decisions, particularly the patients’ qualification for biologic therapy, should include not only the location, extent, and severity of psoriasis lesions but also patient‐reported symptoms, overall disease burden, and limitations in sexual activity and intimate relationships.

## Funding

Eli Lilly provided financial support to the investigator‐initiated study (IIS) “Association of anogenital psoriasis, stigmatization and sexuality—new insights for optimized disease management (PsoGen),” with the protocol I1F‐NS‐O006. Open Access funding was enabled and organized by Projekt DEAL.

## Ethics Statement

This work is part of the project *Association of anogenital psoriasis, stigmatization and sexuality—new insights for optimized disease management* (PsoGen), which was approved by the Ethics Committee of the Hamburg Chamber of Physicians (PV7160, December 10, 2019). The study was conducted in compliance with the World Medical Association Declaration of Helsinki (1964, as revised in 2013), with the criteria of Good Scientific Practice, and with the Standard Operating Procedures (SOPs) of the German Center for Health Services Research in Dermatology (CVderm) on the basis of DIN ISO 9001, 2008; recertified in 2013. All participants provided a signed Informed Consent Form (ICF) in written form.

## Conflicts of Interest

Neuza da Silva Burger, Athanasios Tsianakas, Petra Staubach‐Renz, and Rachel Sommer declare no conflicts of interest. Matthias Augustin has received consulting fees and/or speaker honoraries and/or institutional research support from the following pharmaceutical companies manufacturing drugs for psoriasis: AbbVie, Almirall, Amgen, Biogen, Boehringer, Bristol Myers Squibb, Celgene, Celltrion, Centocor, Eli Lilly, Fresenius, GSK, Hexal, Janssen, Klinge, LEO, MC2, Medac, Merck, MSD, Novartis, Pfizer, Sandoz, Sun, UCB, and Viatris. Dagmar Wilsmann‐Theis has been an advisor, speaker or investigator for Abbvie, Almirall, Amgen, Biogen, Boehringer Ingelheim, Bristol Myers Squibb, Celgene, GlaxoSmithKline, Hexal, Incyte, Janssen‐Cilag, Leo Pharma, Eli Lilly, Medac, Merck Sharp & Dohme Corp., Novartis, Pfizer and UCB Pharma.

## Data Availability

The data that support the findings of this study are available from the corresponding author upon reasonable request.
